# Trajectory of COVID-related tinnitus over the pandemic timeline

**DOI:** 10.1016/j.bjorl.2026.101857

**Published:** 2026-06-16

**Authors:** Ricardo R. Figueiredo, Andréia A. de Azevedo, Guilherme de M.R. Figueiredo, Berthold Langguth, Norma de O. Penido

**Affiliations:** a Centro Universitário de Valença, Faculdade de Medicina, Valença, RJ, Brazil; b Universidade Federal de São Paulo, Departamento de Otorrinolaringologia e Cirurgia de Cabeça e Pescoço, São Paulo, SP, Brazil; c Otorrinolaringologia Sul-Fluminense (Otosul), Volta Redonda, RJ, Brazil; d Universidade Oswaldo Aranha (UNIFOA), Faculdade de Medicina, Volta Redonda, RJ, Brazil; e University of Regensburg, Department of Psychiatry and Psychotherapy, Regensburg, Germany

**Keywords:** Tinnitus, Hearing loss, COVID-19

## Abstract

**Objectives:**

COVID-19 is a respiratory disease caused by the coronavirus SARS-CoV-2. Previous studies reported cases of tinnitus following COVID infection, most of them mild and with similar characteristics to other forms of tinnitus. Our aim is to further explore the relationship between COVID-19 and tinnitus. For this purpose, we analyzed people in our city after a COVID-19 infection.

**Method:**

233 patients over 18-years old and previously confirmed diagnosis of COVID-19 confirmed by either a RT-PCR, antibodies or antigens test were included. Patients were subdivided in three groups: No Tinnitus (NT), tinnitus that already existed before COVID-19 (Chronic Tinnitus, CT) and tinnitus that arose during or after COVID-19 (Post-COVID-19 Tinnitus, PCT). Data concerning COVID-19 symptoms, drugs prescribed for COVID-19, tinnitus characteristics, comorbidities and other otological symptoms were collected. Tinnitus assessment included pure tone audiograms, the Tinnitus Handicap Inventory, visual-analog scales for loudness and distress as well as the hospital anxiety and depression scale. Patients with CT were additionally asked whether their tinnitus worsened after their COVID-19 infection.

**Results:**

No statistical difference was found between CT and PCT concerning hearing, tinnitus characteristics and tinnitus distress. There was also no statistically significant difference between PCT and NT with respect to COVID-19 symptoms and pharmacological COVID-19 treatment. Only 7% of the patients with CT reported worsening of their tinnitus after COVID-19. There was a decrease on post-COVID cases along the pandemic’s timeline: 9.8% in 2020, 6.7% in 2021, 4.4% in 2022 and 0% in 2023 and 2024 (p = 0.058).

**Conclusion:**

Our data demonstrated that PCT tinnitus is very similar to other forms of tinnitus and generally mild. Also, PCT cases decreased along the pandemic’s timeline, possibly due to the onset of vaccination, emergence of omicron as the dominant variant and improvement on the psychological aspects related to the pandemics.

**Evidence level:**

3b.

## Highlights

• Study with 233 adults with COVID-19, divided into tinnitus and non-tinnitus groups.
• Data included symptoms, treatments, THI, VAS, HADS, and audiological findings.
• Post-COVID tinnitus showed distinct features and temporal variation in the pandemic.
• Large clinical cohort revealed prevalence and psychological impact of tinnitus.
• Highlights the importance of monitoring hearing and mental health post-COVID.

## Introduction

COVID-19 is a viral respiratory disease caused by SARS-CoV-2, first reported in China in late 2019 and declared a pandemic by the World Health Organization (WHO) on March 11th, 2020.^1^^,^^2^ The WHO revoked its Public Health Emergency status in May 2023, after more than 776-million reported cases and 7-million deaths worldwide.^3^

Most common symptoms resemble those of other viral infections, but olfactory disturbances were notably frequent during the early waves.^4^ Severe cases could involve respiratory failure, disseminated intravascular coagulation, and cytokine storm.^5^ Over time, multiple variants of SARS-CoV-2 emerged, with the Alpha, Beta, Delta, Gamma, and Omicron variants classified as variants of concern.^6^ Delta and Gamma were more severe, while Omicron, dominant since late 2022, caused milder infections.^7^

Tinnitus, the perception of sound in the absence of external stimuli, affects about 15% of the global population and up to 30% of older adults.^8^^,^^9^ Hearing loss remains the main risk factor.^8^^,^^9^ Several viral infections ‒ including mumps, measles, herpes zoster, and influenza ‒ have been linked to auditory symptoms.^10^^,^^11^ Possible mechanisms include direct cochlear injury, inflammatory response, and drug ototoxicity.^10^

Reported prevalence’s of tinnitus associated with COVID-19 range from 1.2% to 23.2%, though early studies lacked methodological rigor.^12^ Persistent post-infection symptoms, known as “long COVID,” frequently include hearing loss (7.6%), tinnitus (14.8%), and vertigo (7.2%).^13^

Vaccination, which began globally in December 2020, markedly reduced mortality and disease burden. However, tinnitus following COVID-19 vaccination has been reported in 0.038% of mRNA vaccine recipients.^14^ Proposed mechanisms include autoimmune cross-reactivity, nitric oxide reduction, thrombotic effects, ototoxicity, and psychological stress.^15^

In our 2022 publication, we reported that tinnitus arising during or after COVID-19 infection did not differ in its clinical characteristics from tinnitus unrelated to COVID-19.^16^ The present study expands that analysis to include data collected in 2023–2024, examining whether post-COVID tinnitus has changed over time in prevalence or characteristics. By maintaining identical methodological parameters, this study aims to clarify the temporal trajectory of post-COVID tinnitus and its potential evolution across the pandemic.

## Methods

This cross-sectional study was conducted at a private ENT clinic. Between February 2023 and November 2024, all patients attending for any symptom were asked about confirmed COVID-19 infection (RT-PCR, antigen, or antibody tests). Those who answered positively were invited to participate.

Sample size was calculated based on our 2022 study’s post-COVID tinnitus prevalence (19.3%), with a 95% confidence level and 5% error margin, yielding a minimum of 230 participants (McNamar exact test).

Inclusion criteria: adults ≥18-years with confirmed COVID-19. Exclusion criteria: inability or unwillingness to consent.

Data were collected via standardized questionnaires assessing demographics, COVID-19 symptoms, treatments, tinnitus characteristics (VAS, THI),^17^ somatosensory components, comorbidities, habits, and otoscopy. Audiometry followed ASHA guidelines.^18^

### Statistical analysis

Collected data is presented with tables. Numerical data are expressed by central trend and dispersion measurements, and categorical data are expressed by frequency (n) and percentual (%).

Patients were subdivided in three groups: No Tinnitus (NT), patients who already had tinnitus before COVID-19 (Chronic Tinnitus, CT) and patients whose tinnitus developed up to one month after COVID-19 infection confirmation (Post-COVID-19 Tinnitus, PCT), which corresponded to the definition of acute symptom or disease. If patients reported post-Vaccination (VAC) tinnitus, data was collected, but they weren’t included in the analysis.

For the analysis of the possible relationship between drugs used by the patients with the intention to treat COVID-19 and tinnitus development, patients were subdivided in two groups, one including the NT patients and the other including the PCT patients.

Inferential analysis included the following methods:

- For the comparison of numerical data between the three groups (NT, CT and PCT), the Kruskal–Wallis ANOVA (non-parametric) was employed, as well as the Dunn multiple comparisons test (non-parametric). Categorical data were analyzed with the Fisher exact test.
- For comparison between the two tinnitus groups (CT and PCT). The Mann-Whitney test (non-parametric) was employed for the numerical data and the Fisher exact test was employed for categorical data. Non-parametrical tests were applied because the variables didn’t show normal Gaussian distribution, according to the rejection of the normality hypothesis by the Shapiro–Wilk test and graphical analysis of the histogram.

The significance level was established at 5% and the statistical analysis was processed with the 26th version of the statistics software SPSS.

Statistical analyses (Kruskal–Wallis, Mann–Whitney, Fisher’s exact tests) used SPSS v26; p < 0.05 was significant.

As the PCT total cases were very few in the new data, making the analysis of the distribution throughout the pandemic’s timeline unfeasible, and as the settings and methodology of this and our previous study were the same, we included patients from the first 2022 study, only for the analysis of the groups (PCT, CT and NT) distribution. The analysis of the percentual of PCT throughout the years of 2020–2024 was done with the Chi-Squared (χ^2^) test for linear tendency on proportion.

The study was approved by the Ethical Committee. The study was consistent with the Helsinki Declaration for human rights, and all the patients filled in the informed consent after all the study aspects were clarified by the researchers.

## Results

A total of 233 patients were initially included, but two post-vaccine tinnitus cases were excluded, as the aim of this study was to analyze COVID-related tinnitus. So, the final sample included 231 patients. Distribution amongst subgroups NT (No Tinnitus), CT (chronic tinnitus, not related to COVID) and PCT (Post-COVID Tinnitus) is shown on Table 1.

**Table 1:** Numerical variables according to subgroups.

Variable	NT group			CT group			PCT group			p-value
	n	Median	IQR	n	Median	IQR	n	Median	IQR	
Age (years)	168	45.5	33‒63	57	57	43‒67	6	38	25‒52	0.039^1^
Caffeine (mL)	168	100	50‒100	57	100	50‒150	6	125	88‒213	0.030^1^
Tinnitus duration (months)				53	36	7‒120	4	11	3‒24	0.16
VAS volume				57	5	4‒7	4	4	2.5‒7	0.50
VAS distress				57	6	2‒8	4	5	2.5‒7.5	0.91
THI				57	30	13‒50	4	27	15‒43	0.84
HADS anxiety	168	6	4‒9	57	6	3‒11	6	6.5	3.8‒9	0.90
HADS depression	168	4.5	3‒7	57	4	3‒7	6	2.5	0‒6	0.35

Data expressed by the median and Interquartile Range (IQR, Q1‒Q3) and compared with the Kruskal-Wallis ANOVA for three groups or the Mann-Whitney for two groups.

mL, Milliter; VAS, Visual-Analog Scale; THI, Tinnitus Handicap Inventory; HADS, Hospital Anxiety and Depression Scale.

According to the Krukal–Wallis ANOVA, patients in the chronic tinnitus group were significantly older than the other groups and patients in the post-COVID tinnitus consumed more caffeine than the other groups patients. The latter analysis showed the same median for CT and NT groups, but when we considered the average consumption, the averages were 132 mL for the CT group and 94 mL for the NT group.

Considering only the CT group, most patients (78%) didn’t report any changes in their tinnitus, while 7% reported worsening. For 15% of the patients, tinnitus arose after their COVID infection, but the time lapse between infection and tinnitus was too long to be considered PCT. Only 4 out of the 57 CT patients reported worsening of their tinnitus, all of them had only 1 COVID infection, years 2020, 2021 and 2022 (two patients).

Concerning the variables sex, ethnics, comorbities, noise exposure and smoking, there were no statistically significant differences among the goups. Concerning COVID-related data, there was a significant difference on the number of COVID infections, as two infections were most prevalent on the NT and PCT groups, as shown in Table 2. Concerning the year of the first reported COVID infections, COVID symptoms reported and COVID treatment, there was no statistically significant difference.

**Table 2:** Group distribution related to the number of COVID infections.

Variable	NT group		CT group		PCT group		p-value
	n	%	n	%	n	%	
Number of COVID							
One	136	**81.0**	36	63.2	3	50.0	**0.006**
Two	23	13.7	19	**33.3**	3	50.0	
Three	9	5.4	2	3.5	0	0,0	

As for tinnitus characteristics, except for an expected difference towards sudden onset on the PCT group, there were no other statistically significant differences among CT and PCT groups. For the PCT group, most patients (83.3%) reported unilateral constant tinnitus. Most frequently reported types of noise were whistle, followed by hissing, in the CT group and whistle and hissing, equally, in the PCT group.

Concerning the different types of vaccines used, there were no statistically significant differences between the groups. Also, when considering the CT and PCT groups, no statistically significant differences were found concerning the possibility of a somatosensorial component (43.9% on CT group and 0% on PCT group, p = 0.073).

There was a statistically significant difference concerning audiograms between CT and PCT patients. Four out of the six PCT patients had normal audiograms whilst only 26.3% of the CT patients had so. Sensorineural slope audiogram mild hearing loss was the most prevalent type on both the CT and PCT groups.

**Fig. 1:**
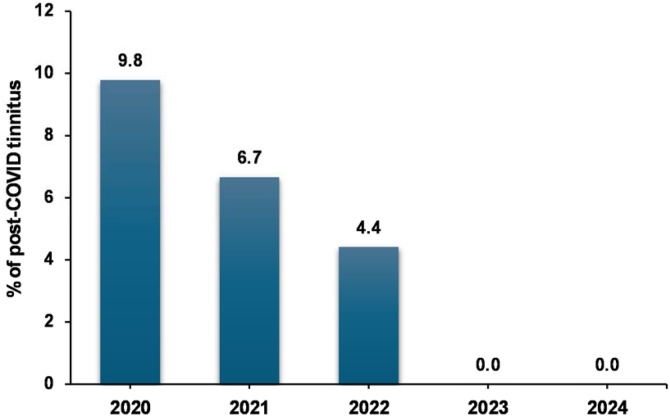
Prevalence of post-COVID tinnitus cases through the years 2020‒2024.

For the analysis of PCT cases along the pandemic’s timeline (from 2020 until 2024), the CT patients were excluded, so the total cases from the two samples was 258 patients. There was a decrease in PCT cases numbers throughout the years, as shown in Fig. 1 (statistically significant tendency, p = 0.058).

## Discussion

The effects of the COVID-19 pandemics were huge, from the sanitary, social, economic, cultural and psychological points of view.^5^ COVID 19-related symptoms reported may be acute or chronic, mild or severe, temporary or permanent, many of them in the otolaryngological domain, and, among those, some audiovestibular symptoms, including tinnitus.^19^

As reported in our first study and other ones, PCT characteristics do not seem to differ from other forms of tinnitus characteristics.^16^^,^^13^^,^^20^ On the other hand, one study reported higher tinnitus severity on post-COVID tinnitus when compared to previous tinnitus.^21^ Most of the PCT patients reported unilateral tinnitus, which is not the most usual, but it failed to reach statistically significant difference when compared to the CT patients, probably due to the very small size of the PCT group (six patients). Also, as well as in our first study,^16^ no difference was found on tinnitus burden, measured by VAS for volume and distress and the THI questionnaire. Most patients reported mild to moderate tinnitus on both groups.

Most studies, including our previous study, reported that COVID didn’t have a significant impact on most of the patients with pre-COVID tinnitus (about 30%–40% of the patients reported that their tinnitus worsened after the COVID infection).^16^^,^^22^ The presented analysis revealed a lower prevalence of worsening (7%), which may be explained by the decrease of COVID-related burden as the pandemics receded.

Additionally, and also in line with our previous study results,^16^ age, comorbidities, habits, tinnitus symptoms and tinnitus treatment did not impact the prevalence of post-COVID tinnitus. Some studies reported an association between inner ear symptoms and olfactory disorders on COVID patients,^23^ suggesting that the affection of the olfactory system increases the risk of auditory involvement, but data from both our studies failed to demonstrate such a relationship.

Our data didn’t show any statistically significant difference on the presence of a somatosensorial component between the CT and PCT groups, but this analysis may have been impacted by the very small size of the PCT group and also by the fact that the “somatosensorial component” was determined by the ENT practioner, not by a trained physical therapist. None of the six patients from the PCT group had a noticeable somatosensorial component.

The association between tinnitus and anxiety/depression is well-known^24^ and was associated with COVID-19-related tinnitus elsewhere.^25^ A 2021 study^26^ demonstrated that anxiety increased among tinnitus patients after the beginning of the pandemic and had an impact on tinnitus burden. Our previous study didn’t analyze this issue with any questionnaire, but, surprisingly, our new data didn’t retrieve significant differences between HADS scores for anxiety and depression between the groups. A possible explanation relies on the fact that most of our data was collected after the mitigation of the pandemics overall burden.

Existent data concerning the prevalence of post-COVID tinnitus are very heterogeneous, ranging from 1.2%^17^ to 23.2%,^27^ and many studies didn’t achieve high quality standards.

A systematic review on this issue concluded that the pooled estimated prevalence of tinnitus following a COVID-19 infection was 8%^28^ and a meta-analysis published by April 2021 reported an event rate of 4.5% for tinnitus among COVID-19 patients.^22^ Our previous data retrieved a somewhat high (19.3%) prevalence of post-COVID tinnitus, but our general clinical impression is that the prevalence of post-COVID tinnitus declined throughout the pandemic’s timeline. To the best of our knowledge, no study analyzed that specifical question.

Considering the limitations of data coming from a private ENT clinic, our new data suggest that post-COVID tinnitus prevalence might, in fact, be decreasing throughout the pandemic’s timeline, as there was a statistical tendency (p = 0.058) to a reduction of the percentual of post-COVID tinnitus from 2020 to 2024. One might speculate about the reasons for such a decline, and we point out three possibilities: (1) The onset of vaccination; (2) The rise of the Omicron variant; (3) The decrease of the general impact of the pandemic on anxiety and depression.

COVID vaccination had a significant impact on the disease morbimortality.^29^ Although many articles have been published concerning the onset of tinnitus after COVID vaccination,^14^^,^^15^ we were not able to find any articles that reported the effects of vaccination on post-COVID tinnitus prevalence. Vaccination in our country started by February 2021,^29^ and our data show a significant decrease on post-COVID tinnitus in 2021 and 2022, when compared to 2020, suggesting a possible impact of vaccination on PCT prevalence reduction.

The omicron (B.1.1.529) variant of concern was first reported in November 2021 in South Africa.^30^ With omicron, several mutations of the spike protein resulted in increased transmissibility when compared to the previous variants, but the infected patients exhibited mild infections and were less frequently hospitalized.^30^^,^^31^ In our data, there was a significant decrease in PCT from 2021 to 2022 (6.7% × 4.4%), and no PCT was reported in 2023 and 2024, allowing us to speculate some role of the onset of Omicron variant as the dominant one on the decrease of PCT prevalence.

COVID-19 pandemics was associated with highly significant levels os psychological distress.^32^ Over the course of the pandemics, disease severity and mortality decreased, due to both vaccination and the emergence of less aggressive virus variants. Accordingly COVID related anxiety decreased as well.^33^ As we mentioned before, the relationship between tinnitus, anxiety and depression is well known and was specifically documented during the pandemic,^22^^,^^28^^,^^34^^,^^35^ so it’s very reasonable to link these findings to the PCT decrease as the pandemics receded.

## Limitations

The study presents a well-structured comparison between Chronic Tinnitus (CT), Post-COVID Tinnitus (PCT), and Non-Tinnitus (NT) groups, offering valuable insights into the clinical and epidemiological characteristics of tinnitus in the context of COVID-19. Strengths include a robust sample size, clear subgroup definitions, and the use of appropriate non-parametric statistical methods. The inclusion of audiometric data, analysis of somatosensory components, and stratification by various demographic and clinical variables contribute to a comprehensive evaluation. Notably, the temporal analysis of PCT prevalence from 2020 to 2024 provides important longitudinal insight, despite the borderline statistical significance (p = 0.058). However, the small sample size in some subgroups, particularly for audiogram comparisons and somatosensory analysis, limits statistical power and generalizability. Additionally, the retrospective design introduces potential recall bias regarding tinnitus onset and COVID-related variables. The lack of psychosocial assessments and longitudinal follow-up data represents a missed opportunity to explore known modulators of tinnitus severity. Finally, the criteria for distinguishing CT from PCT cases are arbitrary. One cannot exclude that tinnitus occurring less than 4-weeks after COVID started without any arousal relationship with the COVID infection, and one cannot exclude that tinnitus starting more than 4-weeks after COVID infection is unrelated to the virus. Despite these limitations, the study adds meaningful data to the evolving understanding of COVID-19's long-term auditory sequelae and point to further directions on the epidemiologic analysis of post-COVID tinnitus prevalence.

## ORCID ID

Andréia A. de Azevedo: 0000-0001-6729-3360

Berthold Langguth: 0000-0002-7066-510X

## Conclusion

Post-COVID tinnitus resembles other tinnitus forms and is generally mild. Most patients with prior tinnitus did not worsen after infection. Somatosensory involvement appears minimal. The prevalence of post-COVID tinnitus decreased from 2020 to 2024, likely due to vaccination, emergence of Omicron, and improved psychological resilience.

## Authors’ contributions

Ricardo R. Figueiredo: Study design; data collection; writing.

Andréia A. de Azevedo: Data collection.

Guilherme de M.R. Figueiredo: Data collection.

Berthold Langguth: Writing.

Norma de O. Penido: Study design; Writing.

## Funding

No fundings for this article.

## Data availability statement

The authors declare that all data are available in repository.

## Declaration of competing interest

The authors declare no conflicts of interest.
